# MFedBN: Tackling Data Heterogeneity with Gradient-Based Aggregation and Advanced Distribution Skew Modeling

**DOI:** 10.3390/s25237314

**Published:** 2025-12-01

**Authors:** Kinda Mreish, Evgenia Novikova, Mikhail Chaplygin, Ivan Kholod, Tarek Alnajar

**Affiliations:** 1 Faculty of Computer Science and Technology, Saint Petersburg Electrotechnical University “LETI”, Saint Petersburg 197376, Russia; mvchaplygin@etu.ru (M.C.); iiholod@etu.ru (I.K.); 2 Faculty of Electrical and Electronic Engineering, University of Aleppo, Aleppo 12212, Syria; 3 Faculty of Electrical Engineering and Automation, Saint Petersburg Electrotechnical University “LETI”, Saint Petersburg 197376, Russia; talnazhar@stud.etu.ru

**Keywords:** federated learning, federated via local batch normalization, non-IID data skews, machine learning, classification, commercial vehicles sensor dataset, NF-UNSW-NB15 dataset

## Abstract

Federated Learning (FL) enables collaborative model training on smart edge devices while preserving data privacy, but it suffers from decreased performance when faced with non-Independent and Identically Distributed (non-IID) data. This paper addresses the problem of the evaluation of aggregation strategies in non-IID FL environments, and it proposes an approach to generation of the skewed datasets with different types of non-IIDness from one dataset: with Feature Distribution Skew; with Label Distribution Skew; with Same Label, Different Features skew; and with Same Features, Different Label skew. The authors also introduce a Modified Federated via Local Batch Normalization (MFedBN), which improves model convergence and robustness across various non-IID data skews by implementing a server-side gradient-style update with several Learning Rate values tested within the aggregated function. Experimental evaluation of the MFedBN strategy was conducted on two heterogeneous datasets, namely, the Commercial Vehicles Sensor dataset designed for monitoring vehicle behavior and the NF-UNSW-NB15 dataset for cybersecurity threat detection. In the majority of cases, the MFedBN algorithm outperformed the baseline FedBN, with test accuracies of up to 85% on the Commercial Vehicles Sensor dataset and 99.98% on the NF-UNSW-NB15 dataset. The model trained with MFedBN showed convergence stability and improved generalization in highly heterogeneous federated environments. The proposed algorithm and data generation methods establish a viable platform for privacy-preserving applications in IoT-based monitoring and network intrusion detection, advancing the validity of Federated Learning in real-world, non-IID conditions.

## 1. Introduction

The communication infrastructure in a smart city integrates a variety of heterogeneous networks, including mobile communication systems, wireless sensor networks, the Internet of Things (IoT), Industrial IoT, and vehicular networks. This distributed and diverse ecosystem, while enabling extensive connectivity, also presents significant challenges to processing and analyzing the volumes of the collected data [1]. Though data volume and availability foster AI development, they also introduce new concerns regarding transmission, storage, computation, and management. In particular, major issues arise when analysis of the distributed data processing is essential but the transmitting of the data to a centralized server is not feasible, due to legal or regulatory restrictions, high communication costs, or technical limitations [2]. As a result, the performance of the AI models decreases due to the lack of necessary data, data bias, or variation in data distribution [3].

Federated Learning (FL) offers a theoretical solution for distributed Machine Learning by enabling collaborative model training without centralized storage of the raw data, thus ensuring privacy of the train datasets and preserving acceptable performance of the local and global Machine Learning models [3]. The key obstacle to the practical application of FL lies in its sensitivity to data distribution across collaborating entities. Although existing FL approaches, such as FedProx [4] and SCAFFOLD [5], have made progress in tackling issues like label distribution imbalance and client drift, they remain less effective when confronted with conditional feature shift and concept shift [6].

In this paper, the authors discuss different types of data heterogeneity or non-IID data skews, and we present an approach to the generation of heterogeneous datasets from one dataset. The most classic FL benchmarks focus on computer vision (CIFAR-10, FEMNIST) and natural language processing tasks, overlooking a vast range of real-world applications that operate on tabular or time series data, such as IoT, cybersecurity, and industrial diagnostics. Our work contributes to this area by providing a solution for generation non-IID subsets for tabular and time series data and performing experiments on two diverse datasets from the cybersecurity and industrial monitoring domains. Using the example of the FedBN aggregation algorithm [7], which was originally developed to tackle the problem of Feature Distribution Skew across the clients, the authors also demonstrate that evaluation of such algorithms’ performance has to take into account different types of heterogeneity. Such comprehensive evaluation not only allows the revealing of algorithm weaknesses but also guides the enhancement procedure of the algorithm robustness.

In this article, a Modified FedBN (MFedBN) strategy is presented. It uses a gradient-style aggregation mechanism in which the global model is updated based on the average deviation of client model updates from the current global state, scaled by a fixed server-side Learning Rate, while the original FedBN follows the standard FedAvg-style aggregation—replacing the global model weights with the weighted average of the client weights for all non-BatchNorm (non-BN) layers. This controlled update approach aims to improve convergence stability and learning effectiveness under non-IID data distributions. Both FedBN and MFedBN aggregation strategies preserve local Batch Normalization (BN) statistics on each client to capture client-specific data characteristics. However, their distinct aggregation styles result in different update dynamics, which can influence convergence behavior and final model performance across heterogeneous client sub-datasets.

Thus, the contributions of this research are as follows:

- an approach to different data skew generation from one tabular or time series dataset;
- a modified version of the FedBN strategy—MFedBN—that uses scaling of the averaged client model updates in respect to a fixed server-side Learning Rate;
- comprehensive experimental evaluation of the original and modified FedBN strategies’ robustness against different data heterogeneity types.

The rest of the paper is organized as follows. In Section 2, we provide the definitions of the data heterogeneity types and we review approaches to processing them during FL training. We also outline open challenges arising due to processing non-IID data. Section 3 presents an approach to the generation of non-IID datasets tailored to the evaluation of aggregation strategies. Section 4 describes the modified Federated via Local Batch Normalization (FedBN) algorithm, detailing its pseudocode and aggregation mechanisms. Section 5 covers our experimental results and analysis, comparing the performance of FedBN and Modified FedBN across different non-IID data skews. Finally, Section 6 sums up the results obtained and defines the direction of our future research work.

## 2. Related Works

Mathematically, the types of data heterogeneity or non-IIDness in FL are defined via joint $P_{i}\left(X,Y\right)$ defined for each client’s dataset $D_{i}$ where *X* refers to the features or attributes in the dataset and *Y* refers to the labels or target classes in the dataset. Joint probability P(X,Y) can be defined as follows: P(X,Y)=P(Y|X)P(X)=P(X|Y)P(Y) , thus allowing us to outline four different types of data heterogeneity [8,9]:

- Feature Distribution Skew or feature shift [7] or covariate shift;
- Label Distribution Skew or class distribution skew or prior probability shift;
- Same Label, Different Features distribution or concept drift;
- Same Features, Different Label distribution or concept shift.

The data skews relating to concept drift and concept shift show in the changes of conditional probabilities P(X|Y) or P(Y|X) , and, therefore, they impact on the decision boundary of the model; these cases are referred to as real concept shift [10,11].

To combat the negative effects of data heterogeneity, a multitude of methods have been proposed, which can be grouped into several key categories:

- aggregation optimization algorithms [4,5,12,13];
- methods based on batch layer normalization [7,14,15];
- model representation alignment [16];
- alternative paradigms [17,18,19,20,21].

**Figure 1:**
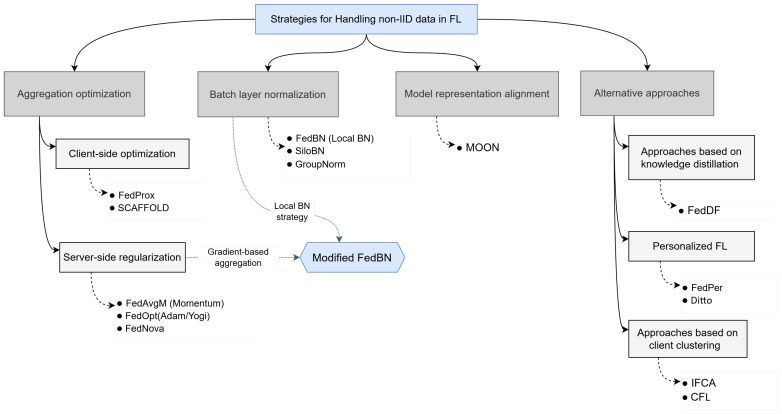
The groups of the proposed approaches to overcoming data heterogeneity problem across clients, including the examples of strategies implementing these approaches: FedProx [4], SCAFFOLD [5], FedBN [7], SiloBN [14], GroupNorm [15], FedAvgM [12], FedOpt [12], FedNova [13], MOON [16], FedPer [17], Ditto [18], FedDF [19], IFCA [20], CFL [21].

Figure 1 shows the main approaches proposed for dealing with data heterogeneity, and examples of such solutions.

The first group of methods aims to directly improve the aggregation process on the server to mitigate the negative effect of client drift, which occurs when local models overfit to their skewed local data. Some approaches address this problem by controlling divergence on the client side: for example, the FedProx aggregation strategy [4] introduces a proximal term to the local objective function to limit the deviation of local models from the global one. Another subclass of strategies in this group uses a server-side optimization step. The FedOpt family of algorithms [12] treats the averaged update from clients as a pseudo-gradient and applies adaptive Learning Rates to it, which helps handle the updates of the varying magnitudes typical in non-IID environments.

The second group of methods solves the problem of the non-IID data on the level of the model’s internal representations, which may reflect the feature skew. It has been shown that Batch Normalization (BN) layers are extremely sensitive to data statistics, which are unique to each client. To combat this problem, excluding BN layer parameters from aggregation while preserving them for local adaptation to each client’s unique statistics has been suggested. This idea was initially implemented in the FedBN strategy [7] and was further developed in SiloBN [14] and the Group normalization approach [15].

The methods in the third group try to align directly the semantic representations of the model. For example, MOON [16] uses contrastive learning to explicitly pull the intermediate representations of the local and global models closer, maintaining their semantic consistency.

Finally, the fourth group consists of approaches that address the non-IID problem by changing the problem formulation itself. It includes solutions that implement the paradigm of Personalized Federated Learning (PFL) [17,18] that focuses on creating personalized models for each client, which is particularly useful in cases of extreme heterogeneity or Same Features, Different Label (SFDL) skew.

Other approaches change the mode of communication. Knowledge distillation methods, such as FedDF [19], evaluate the clients’ exchange outputs (logits) on a public dataset rather than weights, which can be more robust against architectural and statistical differences. Client clustering is another powerful strategy that also avoids constructing the one-model-fits-all assumption. It first identifies groups (clusters) of clients with similar data distributions and then trains a separate, more specialized model for each cluster [20,21,22].

Despite the numerous proposed approaches dealing with non-IID data, this problem is still relevant, as the modeling of real-world non-IIDness data across clients is a challenging task. The majority of the research addresses Label Distribution Skew or Feature Distribution Skew separately, while real-world applications often exhibit multiple simultaneous forms of heterogeneity [23]. The existing standardized frameworks, such as LEAF [24], FedML [25], and Flower [26], provide datasets that simulate different non-IID scenarios. However, the existing protocols have two key limitations that our work aims to address. Firstly, they focus on computer vision (CIFAR-10, FEMNIST) and natural language processing tasks, and they do not provide comprehensive realistic datasets curated for the federated settings for solving tasks from other subject domains. Secondly, the existing methods for generating data skews are limited. The most commonly used approach for simulating label skew is based on the Dirichlet distribution, which parametrically controls the degree of heterogeneity. While a powerful tool, its exclusive use narrows the spectrum of problems studied, especially when it comes to feature skew. To address this issue, alternative approaches have been proposed [27,28]. For example, to generate feature skews the FedArtML tool [28] offers methods based on Gaussian noise to model external interference and the Hist–Dirichlet method to create a controlled one-dimensional shift. However, similarly to LEAF and FedML frameworks, it focuses mostly on transforming image datasets. Thus, there is a *need* in developing methods for generating various realistic non-IID scenarios for tabular and time series data.

This research addresses the identified gap and proposes a novel approach for simulating non-IID FL scenarios with different types of data heterogeneity. It allows the creating of different data partitions with different characteristics from one dataset by redistributing its samples without adding any synthetic noise. The proposed approach does not seek to replace existing approaches but rather to complement them by offering more advanced methods for other types of data and skews, thus contributing to the development of a practical comprehensive evaluation framework for FL algorithms. The authors applied this to evaluating the performance of the proposed Modified FedBN strategy, and we discuss how its application contributes to the comprehensive understanding of the obtained experimental results.

## 3. Approach to Generation of Non-IID Datasets

This section outlines the proposed approaches to the generation of datasets with different types of data skew.

### 3.1. Generation of Datasets with FD Skew

To model Eeature Distribution Skew in a non-IID scenario, two distinct implementations are introduced:

- Variance-Ordered Partitioning for Feature Distribution Skew;
- Wasserstein-Guided Clustering for Feature Distribution Skew.

**Variance-Ordered Partitioning for Feature Distribution Skew**. The key idea of this dataset-partitioning method is to consider the features’ variance and divide the source dataset based on the analysis of the feature with the greatest variance.

Let D={X,Y} be a source dataset where $X={\left\{X_{i}\right\}}_{i=0}^{m}$ is a set of attributes and *Y* is a label, and let $S_{j}=\emptyset$ be a *j* skewed subset that is required to generate from the source dataset *D*, where j=1…n , where *n* is a number of sub-datasets; then, the partitioning procedure is as follows:

1. Calculate variance $\sigma_{i}$ for each $X_{i}$ ;
2. Select attribute $X_{\sigma_{max}}$ with the maximum variance $\sigma_{max}=max\left\{\sigma_{i}\right\}$ ;
3. Sort rows of the initial dataset *D* by sorting the values of the $X_{\sigma_{max}}$ attribute in an ascending manner;
4. Split sorted dataset $D^{sorted}$ into *n* parts with an approximately equal number of rows, i.e., $D^{sorted}={⋃}_{j=1}^{n}\left\{D_{j}^{sorted}\right\}$ ;
5. Assign each part of the split dataset $D_{j}^{sorted}$ to each skewed subset $S_{j}$ : $S_{j}=D_{j}^{sorted}$ .

**Wasserstein-Guided Clustering for Feature Distribution Skew**. The partitioning is undertaken based on the analysis of the feature distribution similarity. The distribution similarity is assessed via the 1-Wasserstein distance (earth mover’s distance). Based on these computed distances, the features are clustered into several groups according to their distributional similarity. Skewed data subsets are then generated by utilizing the extremes of these feature clusters, ensuring that different clients received data with distinct feature distributions.

Let D={X,Y} be a source dataset where $X={\left\{X_{i}\right\}}_{i=0}^{m}$ is a set of attributes and *Y* is a label; and let $S_{j}=\emptyset$ be a *j* skewed subset that is required to generate from the source dataset *D*, where j=1…n , where *n* is the required number of sub-datasets; then, the proposed dataset-partitioning procedure is described as follows:

1. Normalize all features $X={\left\{X_{i}\right\}}_{i=0}^{m}$ ;
2. Compute the matrix of the pairwise distances for all features *X* and cluster them using an agglomerative clustering algorithm into clusters $C_{i}^{X}$ based on computed pairwise distance, i.e., $X={⋃}_{k=1}^{K}\left\{C_{k}^{X}\right\}$ ;
3. Define the minimum ( $x_{i}^{min}$ ) and maximum ( $x_{i}^{max}$ ) thresholds for each feature $X_{i}$ , e.g., by calculating the 25th and 75th quartiles for their values;
4. For each cluster $C_{k}^{X}$ of features:
  - for each feature $X_{l}\in C_{k}^{X}$ , select rows from source dataset *D*, such that its values $x_{l}\in X_{l}$ are less than or greater than the corresponding minimum or maximum thresholds, i.e., $x_{l}\leq x_{l}^{min}$ or $x_{l}\geq x_{l}^{max}$ ;
  - assign selected rows to the subset $S_{j}$ .

### 3.2. Generation of Datasets with SFDL Skew

The generation of the SFDL skew is based on the clustering of the initial dataset in order to obtain data chunks with similar feature distributions, which are then distributed evenly across the required number of skewed subsets. The clustering is performed for features only. To identify the appropriate number of clusters, two internal validation metrics are employed: the Davies–Bouldin score, where lower values indicate better clustering, and the Calinski–Harabasz score, where higher values represent more distinct clusters. Based on these measures, the optimal number of clusters is determined.

Let *D* be a source dataset, and let $S_{j}=\emptyset$ be a *j* skewed subset that is required to generate from the source dataset *D*, where j=1…n , where *n* is the number of sub-datasets; then, the dataset-partitioning procedure is described as follows:

1. Cluster the initial dataset *D* into *K* clusters, i.e., $D={⋃}_{k=1}^{K}\left\{C_{k}\right\}$ .
2. For each cluster $C_{k}$ :
  - split cluster $C_{k}$ into *n* approximately equal parts, i.e., $C_{k}={⋃}_{j=1}^{n}\left\{C_{k}^{j}\right\}$ ;
  - assign each part of cluster $C_{k}^{j}$ to each skewed subset $S_{j}$ : $S_{j}=S_{j}\cup C_{k}^{j}$ .

Through this process, the obtained datasets have comparable feature distributions, while the associated label distributions naturally diverge, thereby representing the same features in a different label non-IID setting.

### 3.3. Generation of Datasets with SLDF Skew

When modeling an SLDF non-IID scenario, the primary focus is on ensuring comparable label distribution across different clients. This can be achieved by dividing the initial dataset based on the values of the label attribute. Then, for each obtained data chunk the attribute with the highest variance is identified, and it is used to sort the chunk in ascending order based on the feature values. These ordered subsets are then divided into smaller, equally sized segments (*n* parts). Finally, these small parts are systematically concatenated to produce subsets with almost identical label distributions.

Let D={X,Y} be a source dataset where $X={\left\{X_{i}\right\}}_{i=0}^{m}$ is a set of attributes and *Y* is a label; and let $S_{j}=\emptyset$ be a *j* skewed subset that is required to generate from the source dataset *D*, where j=1…n , where *n* is the number of sub-datasets; then, the partitioning procedure is described as follows:

1. Split source dataset *D* into subsets based on label value: $D=⋃D_{y_{k}}$ , where $y_{k}\in Y$ .
2. For each subset $D_{y_{k}}$ :
  - calculate variance $\sigma_{i}$ for each $X_{i}$ ;
  - select attribute $X_{\sigma_{max}}$ with the maximum variance $\sigma_{max}=max\left\{\sigma_{i}\right\}$ ;
  - sort rows of the subset $D_{y_{k}}$ by sorting values of the $X_{\sigma_{max}}$ attribute in ascending manner;
  - split sorted subset $D_{y_{k}}^{sorted}$ into *n* parts with an equal number of rows: $D_{y_{k}}^{sorted}={⋃}_{j=1}^{n}\left\{D_{y_{k}}^{sorted_{j}}\right\}$ ;
  - assign each part of the sorted subset $D_{y_{k}}^{sorted_{j}}$ to each skewed subset $S_{j}$ : $S_{j}=S_{j}\cup D_{y_{k}}^{sorted_{j}}$ .

## 4. Modified Federated via Local Batch Normalization (MFedBN) Algorithms

This algorithm represents a variation of the Federated Learning process that incorporates a gradient-style aggregation approach while still applying special handling for Batch Normalization (BN) layers. As in the standard setting, the central server begins by initializing the weights of the global model across all *L* layers, explicitly marking which of these layers are BN layers ( $L_{\mathrm{BN}}$ ). The difference here lies in how updates from clients are integrated into the global model—rather than directly replacing global weights with averaged client weights, the server adjusts the global weights gradually, using a server-side Learning Rate parameter ( η ). Training proceeds over *T* communication rounds.

In each round:

1. The server broadcasts the current global weights $w_{t}$ to all *K* participating clients.
2. Each client initializes its local model with these weights and trains it on its own dataset $D_{t}$ for *E* local epochs with batch size *B*. This step adapts the model to the specific distribution of the client’s local data.
3. After training, each client sends its updated weights and dataset size ( $n_{k}$ ) back to the server.

The server then aggregates the client updates, using two distinct strategies:

- Non-BN layers ( $l\notin L_{\mathrm{BN}}$ ): A weighted average of the client parameters is computed, with each client’s contribution proportional to its dataset size. The resulting update is then scaled by the server Learning Rate ( η ) before being applied to the global model.
- BN layers ( $l\in L_{\mathrm{BN}}$ ): No aggregation is performed; the BN parameters from the previous global model are retained to avoid mismatched BN statistics from heterogeneous datasets.

**Figure 2:**
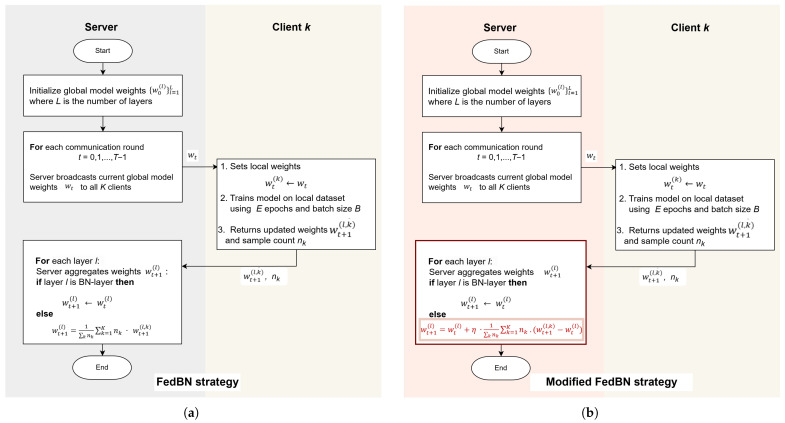
Aggregation algorithms of the (**a**) base FedBN strategy and (**b**) Modified FedBN strategy.

Once updated, the global model is evaluated on a validation dataset to track performance. This process of broadcasting, local training, selective aggregation, and evaluation repeats until convergence or the completion of *T* rounds. Figure 2 shows schematically the algorithms of the base FedBN strategy (Figure 2a) and its novel modified version (Figure 2b); the modified part server side of the algorithm is highlighted by the color, while the red text color shows the exact modification.

### Comparative Analysis of Optimization and Aggregation Strategies

To place the proposed MFedBN strategy within the broader landscape of Federated Learning, it is essential to compare its algorithmic structure with established methods designed for non-IID environments. Table 1 provides a comparative analysis of MFedBN against the baseline FedBN strategy, server-side optimization techniques (FedAvgM, FedOpt), and client-side regularization methods (FedProx), highlighting the fundamental differences in their update mechanisms and parameter sensitivity.

As illustrated, MFedBN distinguishes itself by implementing a lightweight server-side gradient step that dampens update fluctuations without the computational overhead of maintaining the adaptive state vectors required by FedOpt. This approach provides a simpler alternative to client-side regularization while offering more stability than the direct weight replacement used in the original FedBN strategy.

## 5. Experimental Evaluation

To evaluate the proposed aggregation algorithm as well as data-partitioning methods and their impact on FL performance, a series of experiments involving training a deep classifier in a Federated Learning setup with four clients was implemented. All the experiments were conducted in a computing environment with an Intel Xeon 2.0 GHz Ice Lake processor with two cores, supported by 16 GB of RAM and a 300 GB HDD. In terms of software configuration, the experiments were performed using an Ubuntu 22.04.3 LTS operating system. Docker was used to execute the Python 3.10.12-based experimental environment in isolated containers. The Flower Federated Learning framework, the TensorFlow library, and other necessary libraries and dependencies were employed.

In all the experiments, a Deep Neural Network with the same architecture was used. The architecture consisted of an input layer, five fully connected hidden layers with 1024, 512, 256, 128, and 64 neurons, respectively, each incorporating Batch Normalization. The network output layer used a softmax activation function corresponding to the target number of classes. We explored various activation functions, dropout rates, and optimizers—specifically, Adam, Adadelta, and SGD—with the Learning Rate for the Federated Learning aggregation function in MFedBN set between 0.00001 and 0.1, while the Learning Rate for the neural network models in both FedBN and MFedBN was set to 0.0001. The number of server epochs (global epochs) was fixed at 10, while the number of client epochs ranged from 2 to 12 in both FedBN and MFedBN. The final configuration was selected based on achieving the highest test accuracy.

In this work, two distinct datasets were employed to evaluate and validate the proposed methodology. Each dataset captured unique aspects of the target domain, offering diverse data structures, feature types, and classification challenges. The first dataset focused on sensor-based operational data from commercial vehicles in a real-world construction environment, while the second centered on network traffic flows for intrusion detection tasks. Together, they provided a comprehensive testing ground for assessing model performance under different data distributions, feature modalities, and application scenarios.

Section 5.1 and Section 5.2 below provide a detailed description of the selected datasets, the skewed subsets obtained using the proposed techniques, and the performance of the proposed modified aggregation strategy, MFedBN.

### 5.1. Evaluation on the Commercial Vehicles Sensor Dataset

The Commercial Vehicles Sensor dataset [29], donated by Smartilizer Scandinavia AB, consists of numerical measurements drawn from two dump vehicles working at a ground remediation plant near Gothenburg. The purpose of the dataset is to enable analysis and monitoring of industrial vehicle behavior in actual operating conditions. It contains 1,699,983 individual entries, every entry containing the following measurements: timestamp, speed, gyroscope, and accelerometer values, and label, which defines the vehicle state—idle, driving, loading, dumping, and engine-off.

The dataset was split into four parts, using the proposed data-partitioning methods. The characteristics of the obtained datasets are given in Table 2, which shows the heterogeneity in the features and labels distribution across the datasets, measured using the averaged Hellinger distance (HD) and the Wasserstein metric (WM), which is also known as the earth mover’s distance [10]. The Hellinger distance takes values in the interval [0,1] , where 0 refers to identical distributions, and where 1 indicates that two distributions are far apart. Similarly, the Wasserstein metric takes 0 if the probability distributions are identical, and the greater the value of the Wasserstein metric the larger the difference between the probability distributions. The calculation of the averaged distances was implemented as in [28]. Table 2 also clearly demonstrates that it is quite difficult to generate data skews that can be related to only one specific type, as changing distribution in features results in changing distribution in labels. Only for the SLDF distribution is it possible to preserve identical label distribution. The highest heterogeneity in features was obtained for the SLDF method, while the highest heterogeneity in labels was achieved by the SFDL method based on the Wasserstein metric.

**Figure 3:**
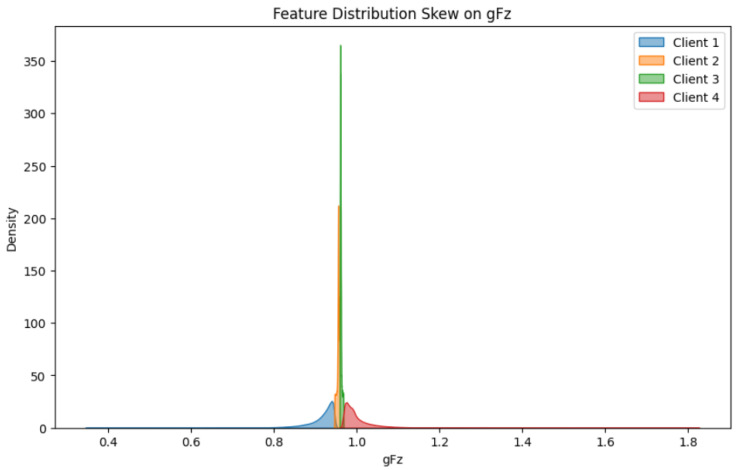
Distribution of the gFz feature across clients in the Commercial Vehicles Sensor dataset.

Figure 3 illustrates the distribution of the feature with the highest variance in the Dumpers dataset—namely, gFz—across four federated clients. The x-axis represents the values of the gFz feature, while the y-axis indicates the density of the observations within each client’s dataset. The kernel density estimates show clear heterogeneity in the way this feature is distributed across the clients.

**Figure 4:**
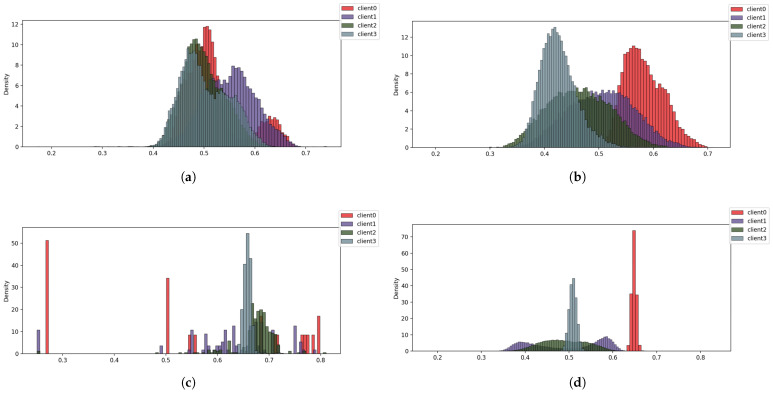
Feature distribution for different classes and different data-partitioning methods: (**a**) class 1 (idle) in the case of FDS-VOP partitioning, (**b**) class 1 (idle) in the case of SLDF partitioning, (**c**) class 5 (engine-off) in the case of FDS-VOP partitioning, (**d**) class 5 (engine-off) in the case of SLDF partitioning.

Although on average the feature heterogeneity level for all five skewed subsets is comparably high, the features for each label (class) are distributed differently due to a different approach to data partitioning. For example, in the case of FDS-VOP the partition was undertaken based on the attribute with the highest variance, while in the case of SLDF the partition was undertaken based on the set of features with the highest variance for each label. This resulted in more diverse feature distribution for each class in the case of the SLDF partitioning. Figure 4 shows the averaged feature distribution for four clients for class 1 (idle) and class 5 (engine-off).

It should also be noted that the Hellinger distance in major cases is equal to 1.0, which does not allow us to judge how different the datasets are. The Wasserstein metric is more sensitive to difference in data distribution, making it more preferable for usage in practice.

**Figure 5:**
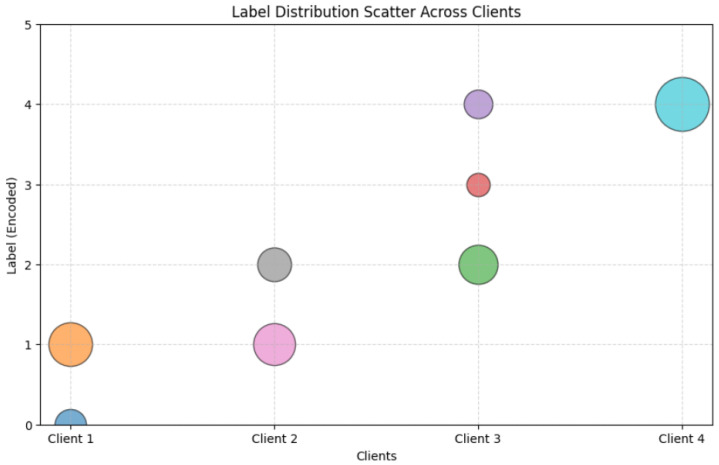
Label Distribution Skew across clients in the Commercial Vehicles Sensor dataset.

The scatter plot in Figure 5 presents the distribution of class labels across four federated clients in the Commercial Vehicles Sensor dataset, obtained using LDS. The horizontal axis denotes the clients, while the vertical axis represents the encoded class labels. Each marker indicates the presence of a particular label in a client’s dataset, while the marker size reflects the relative frequency of that label. The distribution of labels demonstrates clear heterogeneity. Client 1 contained two labels, with a strong predominance of label 1 over label 0. Client 2 was also limited to two labels (1 and 2), but their proportions were more balanced. Client 3 incorporated three labels (2, 3, and 4), although the frequencies were uneven, as suggested by the varying marker sizes. Client 4 was highly skewed, dominated almost exclusively by label 4, which accounted for the majority of its local dataset.

Table 3 shows the performance of the Modified FedBN and original FedBN strategy on the skewed subsets obtained from the Commercial Vehicles Sensor dataset. It can be clearly seen that the Modified FedBN aggregation strategy outperformed the initial FedBN strategy. Only in the case of the SLDF partition does the FedBN show better results; however, they are only slightly better than the results of the random classifier.

The performance of the aggregation strategy depends significantly on the features heterogeneity level. The best accuracy (0.85) was obtained in the case of the FDS-WGC-partitioned datasets, with an averaged Wasserstein metric equal to 0.46. The lowest accuracy of the classifier trained in the FL mode corresponded to the LDS- and SLDF-partitioned datasets, which were characterized by the highest heterogeneity level in features. Moreover, these data partitions had the highest feature heterogeneity within one class across the clients.

**Figure 6:**
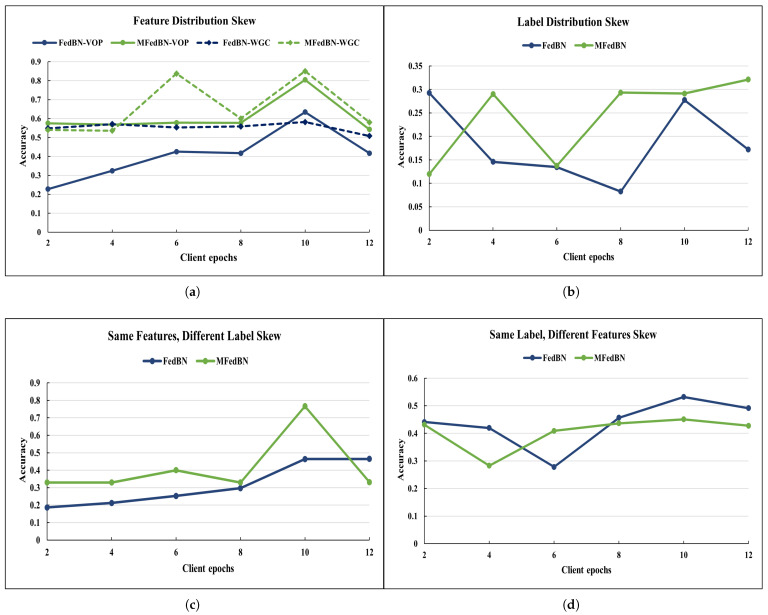
The accuracy of the aggregated model under different types of data skew obtained for Commercial Vehicles Sensor dataset: (**a**) for FDS-VOP-partitioned datasets, (**b**) for LDS-partitioned datasets, (**c**) for SFDL-partitioned datasets, and (**d**) for SLDF-partitioned datasets.

Figure 6 shows how the accuracy of the trained model changed under different types of data skew and aggregations strategies. Although under LDS data skew (see Figure 6a) the accuracy of the model was extremely low, the accuracy of the MFedBN tended to increase with the number of epochs. The reason for such low performance could be the insufficient number of training epochs. In case of the SFDL dataskew (see Figure 6b) the highest accuracy value was achieved for 10 epochs, after which it started decreasing.

**Figure 7:**
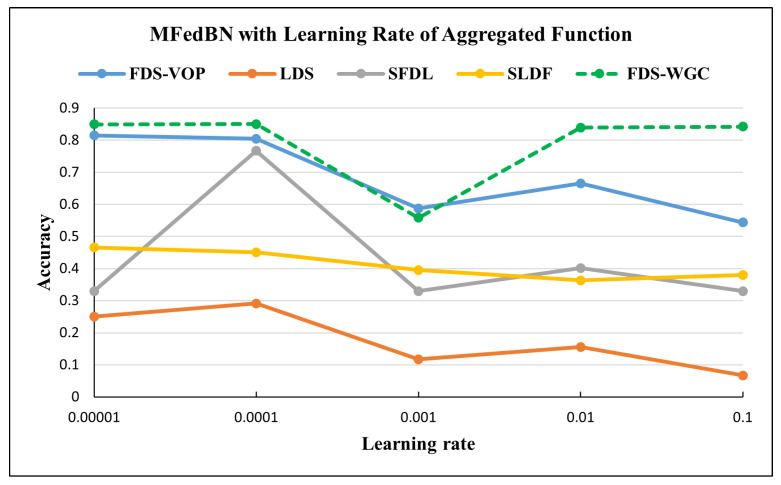
Accuracy of MFedBN with different Learning Rates for aggregated function in the Commercial Vehicles Sensor dataset.

The plot in Figure 7 reports the accuracy of Modified FedBN under the different non-IID skew types and Learning Rates of the aggregated function. For the FDS-VOP-partitioned datasets, the accuracy was high at small Learning Rates (0.8147 at 0.00001, 0.8049 at 0.0001) but dropped to 0.5435 at 0.1, indicating sensitivity to large updates. For the LDS-partitioned datasets with a high heterogeneity level, the accuracy performed the worst, starting at 0.2507 (0.00001) and decreasing to only 0.0669 (0.1), reflecting the difficulty of handling label imbalance. By contrast, for the datasets with moderate feature heterogeneity levels the classifier achieved the most consistent and robust performance, maintaining accuracy above 0.84 across all the Learning Rates.

### 5.2. NF UNSW NB15 Dataset

The NF-UNSW-NB15 dataset is a NetFlow-based adaptation of the well-known UNSW-NB15 benchmark [30], containing a wide range of network attack scenarios. Purpose-built for training and evaluating Machine Learning–driven Network Intrusion Detection Systems, it contains 1,623,118 recorded network flows. Each entry is described through 14 features—a mix of numerical and categorical data—that capture essential network traffic characteristics. The dataset spans 10 distinct traffic classes, from normal Benign flows to malicious categories such as Fuzzers, Analysis, Backdoor, DoS, Exploits, Generic, Reconnaissance, Shellcode, and Worms [31]. In our study, these labeled flows formed the foundation for a distributed neural network model aimed at accurately classifying each subcategory.

Table 4 shows the heterogeneity for feature and label distribution between the datasets, measured using the averaged Hellinger distance (HD) and the Wasserstein metric (WM). Compared to the subsets obtained from the Commercial Vehicles Sensor dataset, the generated subsets were less “heterogeneous”. The maximum feature heterogeneity level was obtained for the FDS-VOP partitioning method, the highest label heterogeneity level for the LDS partitioning skew.

**Figure 8:**
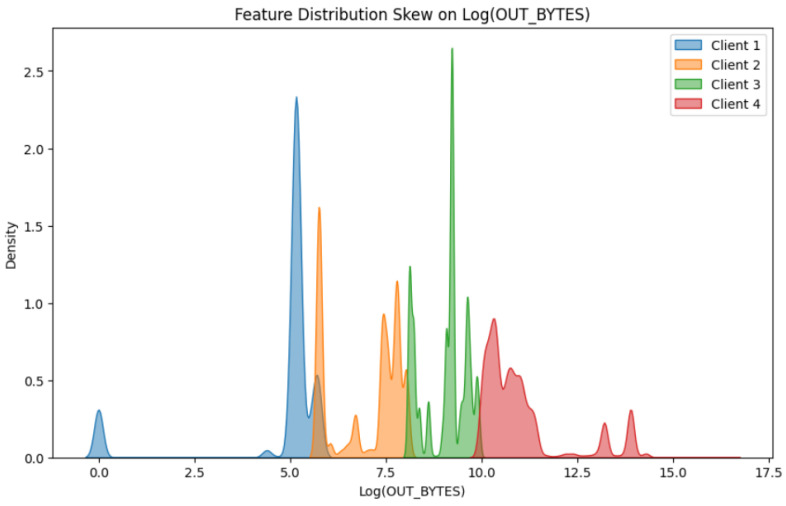
Feature distribution skew of log(OUT_BYTES) across clients in the UNSW-NB15 dataset.

Figure 8 presents the distribution of the feature with the highest variance—namely, the OUT_BYTES feature (in logarithmic scale) across four federated clients derived from the UNSW-NB15 dataset. The horizontal axis denotes the log-transformed values of OUT_BYTES, while the vertical axis represents their estimated density. The feature distribution demonstrates substantial variation among the clients. Client 1 displays a narrow concentration of values centered around log(OUT_BYTES)≈5 , with a secondary accumulation near zero, reflecting predominantly low-to-moderate output traffic. Client 2 is characterized by a distribution that extends between approximately 6 and 8 on the logarithmic scale, with localized fluctuations that indicate moderate variability in traffic patterns. Client 3 exhibits a pronounced concentration around values from 9 to 10, corresponding to heavier traffic volumes and greater consistency within this range. In contrast, Client 4 reveals the broadest spread, extending well beyond 10 and reaching values as high as 15, accompanied by multiple peaks that capture diverse traffic behaviors and substantially higher output volumes.

**Figure 9:**
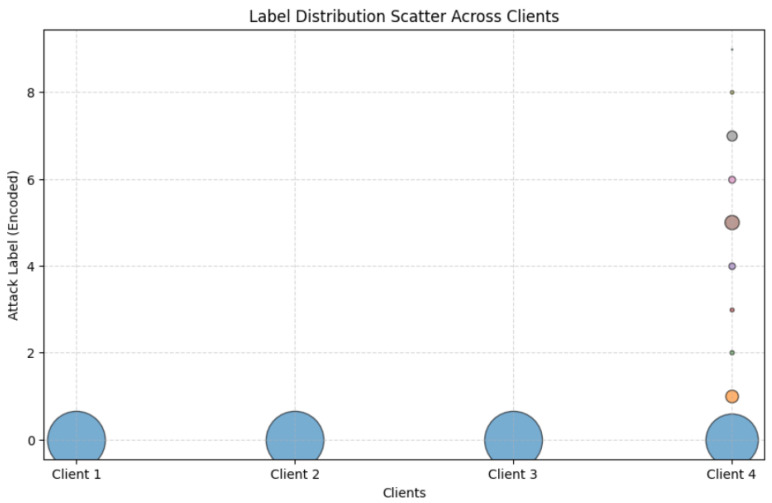
Label distribution scatter plot of labels across clients in the UNSW-NB15 dataset.

Figure 9 illustrates the distribution of the encoded “Attack Label” across four clients in a non-IID dataset. The horizontal axis identifies the clients (Client 1, Client 2, Client 3, Client 4), while the vertical axis represents the encoded label values (0–8). The plot highlights the significant Label Distribution Skew obtained using the proposed LDS partitioning method. Clients 1, 2, and 3 show a tight concentration at label 0, indicating the uniform dominance of this label, with no representation of others. Client 4 exhibits a broader spread, with a primary accumulation at label 0 and secondary points at labels 1 through 8, reflecting a diverse but imbalanced label presence. This heterogeneity underscores the challenge of training a unified model across varied client data distributions.

Table 5 presents the accuracy result achieved by each strategy across different non-IID data cases generated from the NF UNSW NB15 dataset for the best parameter settings. Unlike the Commercial Vehicles Sensor dataset case, the obtained accuracy in all the experiments was high. The performed analysis allowed us to outline the following reasons for such results. The initial dataset was a class-imbalanced dataset; the majority class was represented by a *Benign* class. After partitioning by the proposed methods, the imbalance in classes was preserved, even in the case of the LDS partition (see Figure 9). Thus, the majority class biased the global model towards normal network flow. Moreover, the benign class was well separable from the rest of the classes, which is confirmed by the numerous research papers that use this dataset [32,33].

**Figure 10:**
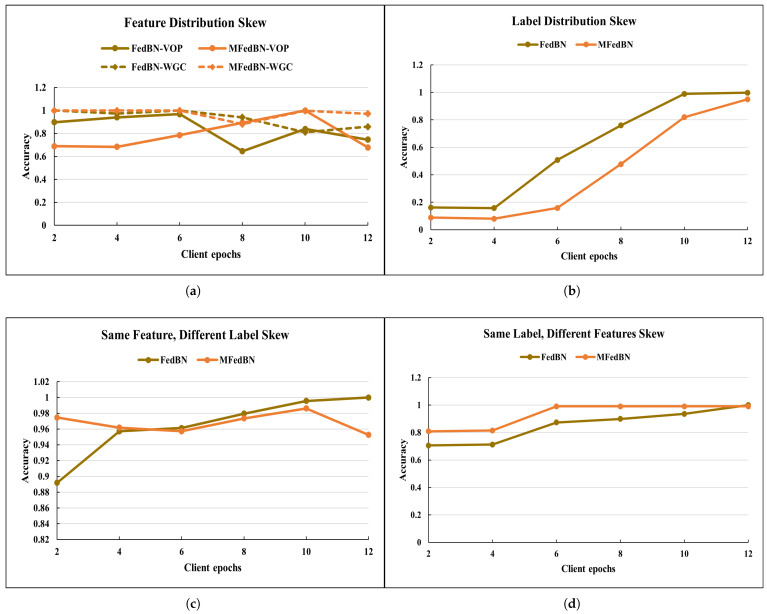
The accuracy of the aggregated model under different types of data skew for the NF-UNSW-NB15 dataset: (**a**) for FDS-VOP-partitioned datasets, (**b**) for LDS-partitioned datasets, (**c**) for SFDL-partitioned datasets, and (**d**) for SLDF-partitioned datasets.

**Figure 11:**
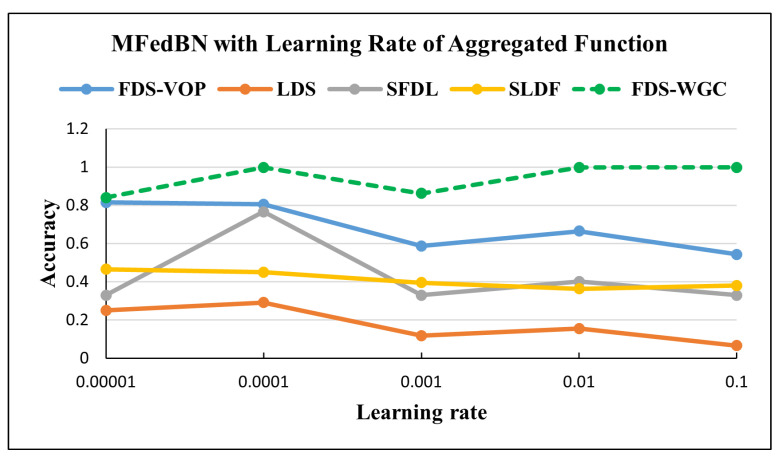
Accuracy of MFedBN with different Learning Rates for aggregated function in the UNSW-NB15 dataset.

Figure 10 reports the accuracy of the Modified FedBN under different non-IID skew types, while Figure 11 shows the dependency of the accuracy on the Learning Rate of the aggregated function. For FDS-VOP partition, accuracy was high at small Learning Rates (0.8147 at 0.00001, 0.8049 at 0.0001) but dropped to 0.5435 at 0.1, indicating sensitivity to large updates. In the case of SFDL data partition, accuracy of the aggregation function showed unstable behavior, with accuracy peaking at 0.7668 (0.0001) but falling back to 0.3299 (0.001).

## 6. Discussion and Conclusions

This study advances Federated Learning by introducing novel methods for generating different types of data skew and a novel MFedBN aggregation strategy, a refined extension of FedBN that employs local Batch Normalization to adapt to client-specific data characteristics and implements a gradient-style aggregation mechanism, scaling averaged client model deviations with a fixed server-side Learning Rate to enhance convergence and resilience against non-IID data challenges.

The proposed set of data-partitioning algorithms allows generating a variety of the data-skewed subsets with different characteristics with quite a high level of heterogeneity, both in features and labels, making it possible to perform comprehensive experimental evaluation of the different aggregation strategies. In contrast to the existing approaches implemented by the most widely used tool, FedArtML, the proposed solution does not modify the original data by adding synthetic noise, but redistributes it. For example, the FDS-WGC data-partition method generates a multivariate shift by operating on the statistical distance between the distributions of entire groups of features, not just the value ranges of a single feature. This allows for a more principled and quantitatively grounded creation of clients whose data distributions are guaranteed to be distinct. We also propose a simpler method, Variance-Ordered Partitioning (VOP), which is effective for creating strong, albeit less nuanced, one-dimensional skews based on the most variant features. For conditional skews, such as SFDL and SLDF data-partitioned methods, we use deterministic partitioning procedures that guarantee that the specified conditional or marginal distributions remain unchanged, allowing for a targeted study of a specific type of heterogeneity. Thus, we expand the toolkit for generating non-IID data. While FedArtML excels at providing a controlled interface for applying known techniques, our work introduces new, statistically grounded methods for generating complex feature skews and conditional skews. These methods are particularly relevant for the often-overlooked but critically important domain of tabular data, where the structure of heterogeneity can differ from that modeled by standard approaches.

A certain limitation of the proposed approach to simulating non-IID scenarios is the lack of a controlling mechanism that allows setting the required degree of heterogeneity. Thus, our future research, in terms of data-partitioning approaches, will relate to the introduction of the mechanism controlling the heterogeneity level, including on the level of the conditional probability of the features and label.

The proposed MFedBN refers to server-side optimization methods; however, it modifies the server step in a fundamentally different way. While the FedOpt family applies complex adaptive optimizers to the averaged update, MFedBN implements a server-side update in the style of a gradient step with a fixed Learning Rate η . Examining the interplay between aggregate accuracy and client local epochs in FedBN and MFedBN alongside variations in the aggregated function’s Learning Rate η in MFedBN reveals nuanced dynamics in handling non-IID skews. The averaged deviation of local model weights from the current global model serves as the update direction (pseudo-gradient). This approach can be interpreted as a conservative update step that dampens sharp fluctuations caused by heterogeneous data by controlling the magnitude of the step via the server-side Learning Rate. The introduced conservative gradient-style update mechanism in MFedBN provides additional gains in stability and accuracy, especially under severe feature skew in comparison to the original FedBN, which uses standard FedAvg strategy for these layers.

Compared to the distillation and clustering methods, MFedBN does not require additional data, nor does it add complexity to the system; thus, it provides a lightweight aggregation algorithm.

The performed experiments show the applicability of the MFedBN to analysis of the tabular data and time series; however, to assess its generality it will be necessary to perform experiments on different types of datasets, including images and texts. Thus, this task defines the second direction of this research. Both directions of our future research will aim at designing a unified testing methodology that will cover not only diverse non-IID scenarios, different data types, but also multiple evaluation metrics and testing scenarios that define base models for each practical task. The current results contribute to this goal by providing necessary tools as building blocks that will form the basis of such a methodology.

## Figures and Tables

**Table 1:** Comparison of MFedBN with baseline, server-side optimization, and client-side regularization strategies.

Feature	FedBN	Modified FedBN (MFedBN)	FedAvgM (Momentum)	FedOpt (FedAdam, FedYogi)	FedProx (Client Regularization)
Core Concept	Addresses feature skew by keeping Batch Normalization statistics local while applying standard averaging to other layers.	Implements a server-side gradient-style update that treats client differences as a pseudo-gradient to dampen sharp fluctuations.	Adds a momentum buffer to the aggregation process to smooth the training trajectory across communication rounds.	Treats the averaged update as a pseudo-gradient and applies adaptive optimizers to handle varying update magnitudes.	Addresses data heterogeneity by modifying the local training objective to explicitly limit the deviation of client models from the global model.
LR Strategy	Uses a fixed Learning Rate on clients, with no additional Learning Rate applied during the server aggregation step.	Uses a fixed server-side Learning Rate ( η ) to scale the aggregated update vector before applying it to the global model.	Uses a server-side Learning Rate combined with a momentum decay parameter to utilize historical update information.	Applies component-wise adaptive Learning Rates on the server, based on the first and second moments of the updates.	Uses standard client Learning Rates but introduces a regularization coefficient ( μ ) to control the strength of the proximal penalty term.
Aggregation Mechanism	Replaces the global model weights directly with the weighted average of the client model weights.	Updates the global model by adding a scaled average of the deviations between the client weights and the current global weights.	Updates the global model using an accumulated velocity vector rather than applying the raw averaged weights directly.	Updates the global model by processing the averaged client updates through an adaptive optimization algorithm like Adam.	Relies on standard weighted averaging at the server level while shifting the burden of handling heterogeneity to the local training phase.
Tuning Sensitivity	Simpler to tune with fewer hyperparameters but may lack convergence stability under severe non-IID shifts.	Highly sensitive to the server-side Learning Rate choice, where smaller values improve stability and larger ones degrade performance.	Requires careful tuning of the momentum coefficient to prevent overshooting the optimal model weights.	Generally more robust against hyperparameter choices due to adaptivity but introduces complex optimizer-specific parameters.	Sensitive to the choice of the proximal term coefficient, which balances the trade-off between fitting local data and staying close to the global model.

**Table 2:** Heterogeneity level of the skewed datasets generated using the proposed methods from the Commercial Vehicles Sensor dataset [29].

Partitioning Strategy	Metric	Heterogeneity Level	
		Features	Labels
FDS-VOP	Hellinger distance	1.00	1.0
	Wasserstein metric	0.56	0.48
FDS-WGC	Hellinger distance	1.00	1.00
	Wasserstein metric	0.46	0.57
LDS	Hellinger distance	1.0	1.00
	Wasserstein metric	0.66	0.73
SFDL	Hellinger distance	1.00	1.00
	Wasserstein metric	0.58	0.75
SLDF	Hellinger distance	1.00	0.00
	Wasserstein metric	0.67	0.00

**Table 3:** Best accuracy results obtained for each non-IID data skew on the Commercial Vehicles Sensor dataset.

FL Strategy	Non-IID Data Skew				
	FDS-VOP	FDS-WGC	LDS	SFDL	SLDF
FedBN	0.63	0.58	0.29	0.46	0.53
Modified FedBN	0.80	0.85	0.32	0.77	0.45

**Table 4:** Heterogeneity level of the skewed datasets generated using the proposed methods from the NF-UNSW-NB15 dataset.

Partitioning Strategy	Metric	Heterogeneity Level	
		Features	Labels
FDS-VOP	Hellinger distance	1.00	1.00
	Wasserstein metric	0.62	0.31
FDS-WGC	Hellinger distance	1.00	1.00
	Wasserstein metric	0.56	0.29
LDS	Hellinger distance	1.00	1.00
	Wasserstein metric	0.30	0.42
SFDL	Hellinger distance	0.67	1.00
	Wasserstein metric	0.22	0.28
SLDF	Hellinger distance	1.00	0.00
	Wasserstein metric	0.55	0.004

**Table 5:** Best accuracy results obtained for each non-IID data skew on the NF UNSW NB15 dataset.

FL Strategy	Non-IID Data Skew				
	VOP-FDS	WGC-FDS	LDS	SFDL	SLDF
FedBN	0.97	0.99	0.99	0.99	0.99
Modified FedBN	0.99	0.99	0.95	0.99	0.99

## Data Availability

The original Commercial Vehicles Sensor dataset presented in the study is openly available in the Kaggle repository at https://www.kaggle.com/datasets/smartilizer/commercial-vehicles-sensor-data-set (accessed on 1 October 2025), and the NF UNSW NB15 dataset is openly available at https://staff.itee.uq.edu.au/marius/NIDS_datasets/ (accessed on 1 October 2025).

## Abbreviations

The following abbreviations are used in this manuscript:

FL	Federated Learning
ML	Machine Learning
IID	Independent and Identically Distributed
LR	Learning Rate
FedBN	Federated via Local Batch Normalization
IOT	Internet Of Things
FedAvg	Federated Averaging
BN	Batch Normalization
NIDS	Network Intrusion Detection System
DNN	Deep Neural Network
FDS	Feature Distribution Skew
LDS	Label Distribution Skew
SFDL	Same Features, Different Label Skew
SLDF	Same Label, Different Features Skew
VOP	Variance-Ordered Partitioning
WGC	Wasserstein-Guided Clustering
